# Beyond the Educational Context: Relevance of Intrinsic Reading Motivation During COVID-19 Confinement in Spain

**DOI:** 10.3389/fpsyg.2021.703251

**Published:** 2021-07-12

**Authors:** Raquel De Sixte, Inmaculada Fajardo, Amelia Mañá, Álvaro Jáñez, Marta Ramos, María García-Serrano, Federica Natalizi, Barbara Arfé, Javier Rosales

**Affiliations:** ^1^Department of Developmental and Educational Psychology, Universidad de Salamanca, Salamanca, Spain; ^2^Estructura de Recerca Interdisciplinar de Lectura, Universitat de València, Valencia, Spain; ^3^Department of Developmental Psychology and Socialization, Università degli Studi di Padova, Padua, Italy

**Keywords:** intrinsic reading motivation, reading behavior, gender, distress, COVID-19

## Abstract

What role could have intrinsic motivation toward reading in an extraordinary situation like the recent confinement? This research examines the relationship between intrinsic reading motivation (IRM) and reading habits in an adult population considering types of reading (for leisure, work/study, social networks, and news), gender, and distress generated by the coronavirus disease 2019 (COVID-19) pandemic. Participants were 3,849 adults from Spain who were surveyed about their reading practices: before, during the first weeks, and after several weeks of confinement. Linear mixed effects models (LMMs) were used to analyze data. Results showed a three-way interaction between reading frequency, IRM, and type of reading. Also, distress seems to pose a differential impact depending on the type of reading. The higher the IRM, the lesser the time devoted to study/work reading and the more to social and news reading (at the beginning of confinement). In this sense, IRM can function as a protective factor of reading behavior but only for leisure reading. Results support previous findings of the importance of consciously promoting this type of motivation in all individuals beyond educational contexts, since it seems to be positively related to well-being. Other results and implications are discussed.

## Introduction

Due to coronavirus disease 2019, the Spanish population was subjected to strict lockdown since March 13, 2020. Recent research has shown how this situation caused many changes in the habits of people, such as eating (Pérez-Rodrigo et al., 2020), use of drugs, TV consumption, time for videogames, and physical exercise (Balluerka et al., 2021). It seems clear that from that moment on, most of the work tasks and whole leisure activities were moved inside the houses, something that had not happened before, greatly altering them in many cases. Reading was one of the daily activities that were most affected by this radical change in habits (Salmerón et al., 2020). Reading habits and the amount of time devoted to different reading activities (or types of reading) are influenced by several individual factors (Scales and Rhee, 2001; Schutte and Malouff, 2007; Garces-Bacsal and Yeo, 2017). For example, there is a positive relationship between intrinsic motivation and time spent reading, a relationship that is stronger for leisure reading (Schiefele et al., 2012). An important research question is whether motives underlying reading habits are maintained in a situation of confinement. This exceptional context offers a unique opportunity to explore the role of intrinsic reading motivation, keeping in mind that the pandemic situation could cause a triggered or emerging situational interest (Linnenbrink-García and Patall, 2016) on certain types of reading. Research on this issue could be beneficial to support the self-determination theory (SDT) and to help us reflect on its potential implications beyond academic contexts. Another individual factor associated with reading behaviors is gender: we know that females have higher levels of intrinsic motivation to read (Wigfield and Guthrie, 1997; Swalander and Taube, 2007; Vansteenkiste et al., 2009) and, therefore, read more than males (Scales and Rhee, 2001). A third individual factor related to changes in habits during the pandemic is individual distress. A situation of confinement and pandemic as the one experienced in 2020 and again in 2021 can generate anxiety and psychological distress (Casagrande et al., 2020), factors that can significantly affect our behaviors (Ingram et al., 2020; Stanton et al., 2020), such as reading habits. For instance, the greater psychological distress experienced during the recent coronavirus disease 2019 (COVID-19) pandemic in China was associated, especially among women, with increased time spent daily on social media searching for and reading information on COVID-19 (Guo et al., 2021). To the best of the knowledge of the authors, the three-way interaction between reading habits, intrinsic reading motivation, and distress has not yet been explored. Thus, the main objective of this study is to understand the nature of such interaction, since this could lead to comprehension of how distress and motivational factors are related to changes in reading habits in highly distressful situations and whether this relationship is different for each gender.

### Motivation (SDT) and Reading

Schiefele et al. (2012) reviewed the construct of reading motivation, which can be understood as current or habitual reading motivation (cf. Pekrun, 1993). In this research, we focused on the habitual reading motivation which “… denotes the relatively stable readiness of a person to initiate particular reading activities” (Schiefele et al., 2012, p. 429). Regardless of this distinction, Schiefele et al. (2012) also provided a comprehensive review of the dimensionality of the construct of reading motivation. From a psychological perspective, it has been studied from different lines of research, for instance, through the value beliefs of reading tasks (e.g., Durik et al., 2006; Solheim, 2011), from the self-concept and self-efficacy of reading (e.g., Chapman and Tunmer, 1995; Wigfield and Guthrie, 1997; MRQ reading efficacy subscale) or *via* orientations of objectives related to reading (e.g., Graham et al., 2008). However, according to Schiefele et al. (2012), the most important distinction is the one that exists between intrinsic and extrinsic reading motivation. Intrinsic reading motivation is defined as the willingness to read because the activity is satisfying in itself for an individual. In contrast, extrinsic reading motivation refers to when reading is motivated by its expected consequences, either to obtain positive outcomes or to avoid negative ones (Wigfield and Guthrie, 1997; Becker et al., 2010; de Naeghel et al., 2012, 2014; Schiefele et al., 2012, 2016). These types of reading motivation can be described as higher order categories subsuming several dimensions.

The SDT (Ryan and Deci, 2019) is an exceptional framework for approaching the study of these motivational categories. As an organismic theory, SDT posits that its representation of motivation is universal (Pintrich and Schunk, 2006; Ryan and Deci, 2020). This is justified by considering the existence of three basic psychological needs that are innate to human behavior: autonomy, competence, and relatedness. Autonomy refers to the basic psychological need of feeling as the initiators of our own actions, so it is promoted by experiences of personal interest and value (Ryan and Deci, 2020). Externally controlled experiences, such as rewards and punishments, may undermine autonomy. Individuals also need to feel competent in their interactions with other people, in the tasks and activities they perform, and in adapting to their environment in a broad sense. This need is best satisfied when tasks or activities afford optimal challenges and opportunities for growth. Finally, relatedness refers to the need of being part of a group, so it has commonly been denominated as a need for belonging (Pintrich and Schunk, 2006; Ryan and Deci, 2020). It is promoted by respect and affection. Taking these needs into account, intrinsic motivation can be defined as “the human need of being competent and self-determined in relation with the environment” (Deci, 1980, p. 27). Autonomy and competence needs are, thus, directly related to intrinsic motivation (Garon-Carrier et al., 2016).

From this theoretical perspective, most of the general outcome categories (i.e., achievement, persistence, well-being) treated in motivational research are not exclusive to the education domain. Hence, “…current results in this research field can be probably generalized to domains beyond education such as workplaces, health-related behavior and interpersonal relationships” (Howard et al., 2021, p. 19). For this reason, SDT can serve as a framework for studying adult reading habits.

According to SDT, the types of reading motivation can also be ordered on a continuum of self-determination (Howard et al., 2017; Ryan and Deci, 2020), varying from an absence of self-determination (i.e., motivation), to partially self-determined behaviors (e.g., an extrinsic motivation referred to as introjected regulation), to the most self-determined behaviors (i.e., intrinsic motivation). Thus, intrinsic motives will always be self-determined, whereas extrinsic motives can be categorized as more or less self-determined: external, introjected, identified, and integrated regulation (Ryan and Deci, 2019, 2020; Howard et al., 2021). External regulation concerns behaviors driven by externally imposed rewards and punishments (e.g., reading to avoid punishment). Introjected regulation is characterized by ego-involvement motives, because the goal is to gain and maintain approval from the self and others (Howard et al., 2021), driven by feelings of “I should…” and guilt. Identified and integrated regulations are the most self-determined of these extrinsic motives. Individuals are driven by an identified regulation when their behaviors are consistent with perceived personal values and meaning, regardless of the enjoyment derived from enacting those behaviors. Finally, when driven by an integrated regulation, individuals assimilate the enactment of a behavior into their sense of self such that the behavior becomes a fully congruent element of their identity. One of the core hypotheses of SDT is that supporting basic psychological needs facilitates the most autonomous types of motivation (i.e., intrinsic motivation and integrated and identified regulation), whereas thwarting those needs undermines it (Ryan and Deci, 2020).

This study focuses on the most self-determined type of motivation: intrinsic reading motivation. This decision is based on the strength of this type of motivation to predict performance and well-being, making it possible to consider it as the most optimal type of motivation (Garon-Carrier et al., 2016). A very recent meta-analysis on SDT by Howard et al. (2021) has reinforced this argument and has provided revealing data linking intrinsic motivation with some individual variables related to well-being (e.g., anxiety and distress), a relationship that we intend to explore in this study.

Intrinsic motivation can be defined as a psychological desire to execute behaviors (reading, in this case) for the only purpose of obtaining the satisfaction, pleasure, or excitement associated with enacting the behavior itself (Ryan and Deci, 2019). In other words, reading is intrinsically motivating as long as it satisfies the psychological needs of competence and autonomy (Ryan and Deci, 2009).

In this study, we expected the confinement to have an impact on how people met their basic needs through their reading habits. We also explored the association between intrinsic reading motivation (IRM) and reading behavior (i.e., reading frequency) and how it varied with the type of reading, gender, and distress in such an exceptionally stressful context. The results obtained can offer valuable information about their relevance beyond an educational context in the adult population.

### Intrinsic Reading Motivation, Reading Behavior, Type of Reading, Gender, and Distress

Studies have consistently found a positive relationship between intrinsic reading motivation and reading behavior (Wang and Guthrie, 2004; Unrau and Schlackman, 2006; Law, 2008, 2009; Becker et al., 2010; Retelsdorf et al., 2011). Hence, people who read for their own enjoyment read more often than those who read for other external motivations, such as to avoid a punishment or to obtain a reward. However, this association has been mainly observed in educational contexts. Schiefele et al. (2012) suggested how in educational settings the association between intrinsic motivation and reading frequency is stronger for leisure reading than for academic reading, finding that the higher the intrinsic motivation to read, the higher the frequency of leisure reading. Furthermore, de Naeghel et al. (2012) found that, regardless of the context in which motivation is measured (i.e., recreational versus academic), autonomous motivation is positively related to leisure reading frequency. Studies focused on adult reading motivation are rare. Schutte and Malouff (2007), although did not specifically analyze intrinsic motivation, found that understanding reading as part of the self (reading as part of one’s identity or, according to SDT, self-determined motivation) was strongly related to reading frequency (self-reported measure). This was moderately related to hours spent on leisure reading and not significantly associated to hours spent on mandatory reading.

Previous studies have also found that gender plays an important role in the relationship between reading motivation and frequency and/or type of reading. Studies focused on reading motivation find that girls are more intrinsically motivated to read, and this is positively associated to reading frequency (de Naeghel et al., 2012). Moreover, higher intrinsic motivation toward academic tasks of girls is positively related to their achievement and learning (Ratelle et al., 2007; Vansteenkiste et al., 2009). Also, the studies focused on adult reading (Schutte and Malouff, 2007) have found that women scored significantly higher than men on reading motivation scales and on self-determined motivation subscales. In addition, studies and surveys on reading habits systematically show that women read more than men, and especially read more for leisure (Scales and Rhee, 2001; Mellard et al., 2007; Federación de Gremios de Editores de España (FGEE), 2020) even in confinement (Salmerón et al., 2020; Federación de Gremios de Editores de España (FGEE), 2021).

The results on motivation to read and gender effects seem quite robust. However, in such an exceptional situation as confinement, there might be other factors that could affect the relationship between motivation, gender, and reading habits. Casagrande et al. (2020), for example, have found that the COVID-19 outbreak has had a psychological impact in terms of anxiety symptomatology and psychological distress on a population of 2,291 Italian respondents to a survey. Similarly, Alzueta et al. (2021) analyzed the psychological impact of the pandemic across 59 countries around the world. They found that a significant proportion of respondents reported moderate to severe symptoms of depression (25.4%) and anxiety (19.5%), and that European citizens, women, and young adults were the ones who obtained higher scores. Experiencing psychological distress is significantly associated with changes in health and physical behavior (Ingram et al., 2020; Stanton et al., 2020). Moreover, Guo et al. (2021) suggested an association between distress and reading behaviors. Concretely, they found that the amount of time people spent daily on social media searching for and reading information on COVID-19 is, among other things, a predictor of psychological distress. Also, Alzueta et al. (2021) suggested that younger adults may be more vulnerable to the psychological effects of the COVID-19 pandemic as a consequence of greater exposure to media. However, a systematic analysis of how distress generated by confinement might affect reading habits is, to the best of the knowledge of the authors, still lacking. Such association may also be influenced by motivational factors: we know from previous studies that people who have high intrinsic reading motivation toward learning tasks (i.e., musical or academic content) experience less distress in task performance than those with less self-determined motivation (Stoeber and Eismann, 2007). That is, it seems that being intrinsically motivated toward a learning task protects against the occurrence of distress associated with the performance of that task. Furthermore, Levine et al. (2020) found that students with autonomous motivation read more books recreationally, which was associated with lower levels of psychological distress. They conclude that recreational reading mitigates the frustration of one’s basic psychological needs by improving the feeling of being autonomous, competent, and socially connected. Thus, exploring the association between distress, reading habits, gender, and motivation is important to better understand the role of intrinsic motivation on reading. It is possible that people with high levels of intrinsic motivation toward reading do not vary their reading frequency even though they experience a high degree of distress generated by the confinement situation (Levine et al., 2020). However, according to Guo et al. (2021), the time spent on social media and reading news about COVID is a predictor of distress, so we wonder whether the IRM would play some role in this relation, as reasons of people for reading social media and news might be independent of IRM.

Therefore, one of the challenges we sought to address in this correlational study is to explore whether these relationships between intrinsic reading motivation and reading frequency are maintained in the adult population from prior to confinement, at the start of confinement, and after a month of confinement.

### Hypotheses

Based on prior studies, we made the following hypotheses:

H1: IRM will be related to reading frequency (RF). Specifically, it is expected:

H1.1: A positive relationship between IRM and RF, so that those people who report higher IRM will show higher RF than those who report low intrinsic motivation (Schiefele et al., 2012).

H1.2: This positive relationship between IRM and RF will occur at the three points in time investigated: prior to confinement, at the start of confinement, and during the continuation of confinement.

H1.3: According to previous studies (de Naeghel et al., 2012), we did expect to find a positive relationship between IRM and RF in the types of academic or work and leisure reading, a relationship that will be stronger in the latter (de Naeghel et al., 2012, 2014). On the other hand, we did not expect a specific relationship between IRM and social reading and reading news. It can be hypothesized that news and social reading may not be the main means of satisfying the pleasure associated with enacting the reading behavior itself.

H2: IRM and RF will be different for males and females. Differences will be the following:

H2.1: Based on previous studies, we expect females to have higher IRM than males (e.g., Wigfield and Guthrie, 1997; Swalander and Taube, 2007; Vansteenkiste et al., 2009). Therefore, and in line with hypothesis 1.1, females will spend more hours reading (RF).

H2.2: The superiority of women in terms of intrinsic motivation and reading frequency will be constant across the three-time period: before, in the beginning, and few weeks of confinement.

H3: According to previous studies (Casagrande et al., 2020), situations of confinement can generate stress and overloads in individuals. We expected that these stressors will condition to a lesser extent the RF in people with higher IRM (Howard et al., 2021), especially in the type of leisure reading (Levine et al., 2020).

## Design

### Participants

The sample for this study was formed by 4,181 adults from Spain. From that initial sample, 332 participants were excluded because of one or several of the following reasons: they did not accept the use of their data for the study, they did not currently live in Spain, they showed an incoherent pattern of responses, or they were not in the 18–65 age range (see Salmerón et al., 2020, for more information on the database used for this study). After removing all these cases, the final sample was composed of 3,849 Spanish adults between 18 and 65 years old (*M* = 33.52 years, *SD* = 13.93). As shown in Table 1, the final sample was predominantly female, young, and medium-educated.

**TABLE 1:** Descriptive statistics of the participants.

***N***		**3849**
Gender	Female	2724 (70.8%)
	Male	1125 (29.2%)
Age range	From 18 to 24 years	1620 (42.1%)
	From 25 to 34 years	683 (17.7%)
	From 35 to 44 years	548 (14.2%)
	From 45 to 54 years	567 (14.7%)
	From 55 to 65 years	431 (11.2%)
Occupation	Students	1782 (46.3%)
	Workers	1944 (50.5%)
	Unemployed	222 (5.8%)
	Retired	79 (2.1%)
	Other	28 (0.7%)
Completed studies	Primary education	32 (0.8%)
	Secondary education	1858 (48.3%)
	Undergraduate degree	1200 (31.2%)
	Master degree	534 (13.9%)
	Ph.D. degree	220 (5.7%)
	Other	5 (0.1%)

*Percentages in brackets are shown.*

A non-probabilistic sample was used. A link to the survey was published online and sent to educational institutions, friends, and family members asking to forward it. In order to facilitate the generalization of the results, a weighting adjustment method was performed to account for misrepresented groups. First, the reference values (i.e., frequencies by age and gender), as provided by the Spanish Nation Institute of Statistics (Instituto Nacional de Estadística, 2020), were computed. Then, the observed frequencies by age range and gender in the database were computed. The weight value was *W* = (n_*p*_/n_*s*_) × (N_*s*_/N_*p*_), where n_*p*_ is the frequency by age and gender in the population, n_*s*_ is the frequency by age and gender in the sample, N_*s*_ is the sample size, and N_*p*_ is the population size.

### Procedure

All data were extracted from the database READ-COGvid (Salmerón et al., 2020), collecting from April 11, 2020 to April 19, 2020 in Spain.

Data for the READ-COGvid reading habits survey were collected by an unrestricted self-selected survey. It published a link to the survey on social media and sent links to the survey to educational associations, undergraduate students from several universities in Spain, and to social networks of the researchers with a request to spread it. Responses were collected *via* the tool LimeSurvey, and data were stored in the servers of the University of Valencia, following the GDPR Compliance. The study was designed following the ethical principles of the Declaration of Helsinki. Before their participation, the participants were informed about the goals of the study and about the ethical guidelines followed in the design and data treatment.

### Measures

#### Intrinsic Reading Motivation

An adapted version of the SRQ -Reading Motivation Questionnaire (de Naeghel et al., 2012) was used, developed to capture intrinsic reading motivation (e.g., “I read because I enjoy reading”). Four items were selected from the original questionnaire. Items were scored on a five-point Likert scale, ranging from 1 (completely disagree) to 5 (completely agree). The four-item questionnaire had good internal consistency: McDonald’s omega (ω = 0.89) and average variance extracted (AVE = 0.65). A confirmatory factor analysis (CFA) was performed to assess the reliability of the instrument more deeply. Because of the ordinal nature of the scale, the diagonally weighted least squares (DWLS) estimation was selected. The factor model was significant (χ^2^ = 37.879; *p* < 0.001). Additional fit measures were appropriate: IFI = 0.984, GFI = 0.993, CFI = 0.984, RMSEA = 0.068.

#### Reading Frequency on Type of Reading

This scale assessed how much daily time the participants recalled spending on different reading activities: reading for leisure, reading for work or study, reading news to keep up with current events, and social reading (Scales and Rhee, 2001; Torppa et al., 2020). For each reading activity, they answered using the following scale: nothing, ∼30 min a day, 1, 2, 3, 4 h a day.

#### Reading Habits Times

The participants fulfilled the previous scale (reading frequency on type of reading) three times: first, they recalled the last time (before the confinement) they had spent few days at home (e.g., holiday, weekend…), and that report was used for the “Before” measure. Then, they fulfilled the scale again recalling the first 2 weeks of confinement (this was the “Beginning” measure). Finally, for the “Month” measure, they reflected about the current period, after few weeks of confinement had passed. Therefore, the survey was sent just once to each participant, and they fulfilled the reading frequency on the type reading scale three times, just recalling different points in time.

#### Distress

The personal distress (PD) subscale of the Interpersonal Reactivity Index (IRI) (Davis, 1980) was used to assess the distress of the participants during the lockdown. This is a seven-item five-point Likert scale tapping feelings of anxiety and self-control in tense situations. The respondents rated statements such as “In emergency situations, I feel apprehensive and ill-at-ease” from 1 (does not describe me well) to 5 (describes me very well). Validated adaptation in Spanish (Escrivá et al., 2004) was used. The PD scale showed good internal reliability in the sample (ω = 0.82; AVE = 0.42). Although AVE is a bit low, it is still considered appropriate if composite reliability is higher than 0.6 (Fornell and Larcker, 1981). Composite reliability for the subscale is 0.84. A CFA was performed to assess the reliability of the instrument more deeply. Because of the ordinal nature of the scale, the DWLS estimation was selected. The factor model was significant (χ^2^ = 548.527; *p* < 0.001). Additional fit measures were appropriate: IFI = 0.912, GFI = 0.973, CFI = 0.912, RMSEA = 0.1. Although RMSEA is a bit high, this test is not very reliable on its own, and previous studies have suggested that there is no support for universal RMSEA cut-off points, recommending the use of the other goodness of fit measures to make decisions (Chen et al., 2008). Overall, the scale reliability is fair.

### Data Analysis

In order to test H1 and H2, we used a linear mixed effects model (LMM) with the lmer and emmeans packages in R (R Core Team, 2020) for each type of reading (leisure vs. news vs. social vs. study/work), which is four in total. The categorical fixed factors were time (before vs. beginning vs. month) and gender (female vs. male), and the continuous fixed factor was IRM. The participants were the random effect in the model. The dependent variable was the number of reading hours per day (reading frequency, RF). As RF was not normally distributed, before performing any statistical analysis, we log transformed it, which resulted in more normal distributions. The rest of the assumptions for the analysis were met, the *F-* and *p*-values for the analyses. For each significant effect, we also report in-text, the β values (standardized coefficients) as a measure of effect size (β < 0.2 = weak, β > 0.2 and <0.5 = moderate, β > 0.5 = strong effect, Acock, 2014). The effect size was calculated with the function “effectsize: standardize_parameters()” in R.

## Results

### Effect of Intrinsic Reading Motivation, Time of Confinement, and Type of Reading on Reading Frequency

A table with all the effects for each type of reading can be seen in Table 2. For clarity purposes, results will be commented for each type of reading next.

**TABLE 2:** Effects of IRM, time, and gender on RF for each type of reading.

**Study/Work**						
	**Sum sq**	**Mean sq**	**NumDF**	**DenDF**	***F* value**	**Pr(>F)**

Time	0.08	0.04	2	8305.9	0.37	0.692
IRM	2.64	2.64	1	3991.8	24.18	0.000***
Gender	0.03	0.03	1	4078.4	0.25	0.614
Time:IRM	0.4	0.2	2	8305.9	1.82	0.162
Time:Gender	0.47	0.24	2	8305.9	2.18	0.114
IRM:Gender	0.03	0.03	1	3991.8	0.26	0.607
TIME:IRM:Gender	0.82	0.41	2	8305.9	3.75	0.024*
**Leisure**						
Time	0.25	0.13	2	8189.6	1.56	0.210
IRM	46.48	46.48	1	3793.5	576.63	<2.2e-16***
Gender	0.04	0.04	1	3886	0.53	0.465
Time:IRM	1.52	0.76	2	8189.6	9.41	0.000***
Time:Gender	0.27	0.13	2	8189.6	1.67	0.189
IRM:Gender	0.06	0.06	1	3793.5	0.76	0.384
TIME:IRM:Gender	0.17	0.09	2	8189.6	1.07	0.342
**Social**						
Time	0.6	0.1	2	7902	8.91	0.000***
IRM	2.41	2.41	1	3766.2	71.87	<2.2e-16***
Gender	0.79	0.79	1	3822.2	23.49	0.000***
Time:IRM	0.14	0.07	2	7902	2.07	0.126
Time:Gender	0.01	0.01	2	7902	0.21	0.812
IRM:Gender	0.22	0.22	1	3766.2	6.57	0.010*
TIME:IRM:Gender	0.01	0.00	2	7902	0.08	0.924
**News**						
Time	0.55	0.28	2	8023.8	5.16	0.006**
IRM	0.86	0.86	1	3596.8	15.97	0.000***
Gender	0.03	0.03	1	3689.4	0.57	0.449
Time:IRM	0.08	0.04	2	8023.8	0.75	0.474
Time:Gender	0.04	0.02	2	8023.8	0.38	0.685
IRM:Gender	0.03	0.03	1	3596.8	0.63	0.427
TIME:IRM:Gender	0.02	0.01	2	8023.8	0.16	0.849

*Sig. codes.* 0***; 0.001**; 0.01*. The data reported in this table have been calculated with transformed reading frequency (RF).

#### Leisure Reading

Table 2 shows that the main effects of time of confinement and gender were not significant. However, the main effect of IRM was significant. As predicted, the higher the IRM, the higher the RF (β = 0.29). The interaction between time and IRM was also significant: although the relationship between intrinsic motivation and RF was positive at any moment of confinement, it was stronger after a month of the confinement than at the beginning and before (χ^2^ = 46.477, *p* < 0.001); that is, the slope of IRM was higher at the month of confinement (β = 0.06).

#### Study

In the case of reading for study/work, only the main effect of IRM was significant (β = 0.06); that is, the higher the IRM, the higher the RF, so H1.1 was confirmed. The effect of time was only significant in interaction with IRM and gender, so we will analyze it in the next section.

#### News

**FIGURE 1:**
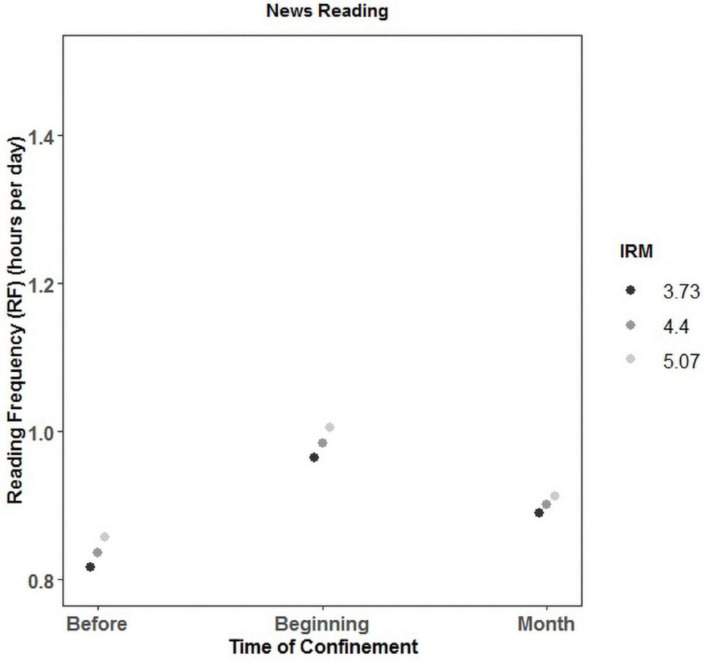
Interaction between reading frequency (RF), time, and intrinsic reading motivation (IRM) for news reading.

In the case of news reading, the main effect of IRM was again significant; that is, again, the higher the IRM, the higher the RF (β = 0.05), confirming H1.1. The main effect of time was also significant, but in this case, the RF was significantly higher at the beginning of the confinement (β = 0.39) than before and after a month (β = 0.33) (see Figure 1) regardless of the level of intrinsic motivation. The interaction between time and IRM was not significant.

#### Social

**FIGURE 2:**
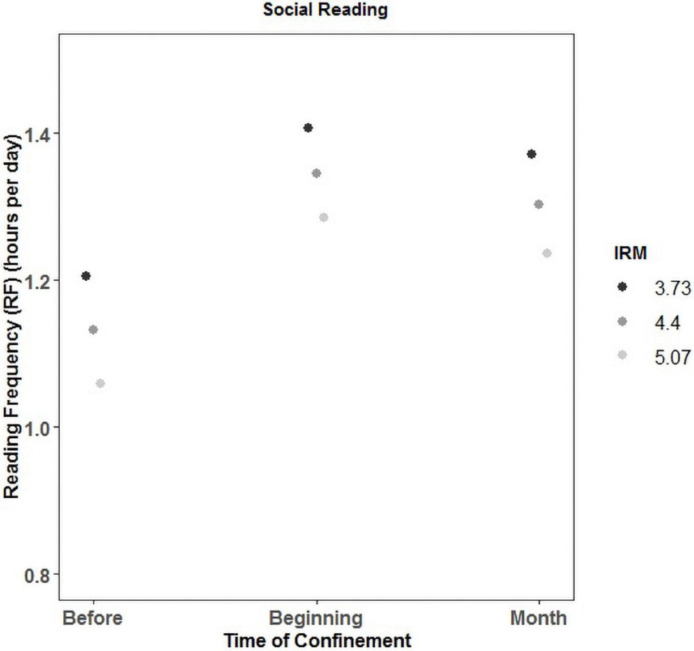
Interaction between reading frequency (RF), time, and intrinsic reading motivation (IRM) for social reading.

Finally, in the case of social reading, the main effect of both IRM and time was again significant. However, in the case of IRM (β = −0.18), contrary to the pattern in the rest of types of reading, the higher the IRM, the lower the frequency of social reading (before: χ^2^ = 70.146, *p* < 0.001; beginning: χ^2^ = 47.8; *p* < 0.001; month: χ^2^ = 61, *p* < 0.001). The effect of time followed the same pattern than in the case of news, that is, the RF was significantly higher at the beginning of the confinement (β = 0.33) than before and after a month (β = 0.33) (see Figure 2) regardless of the level of intrinsic motivation.

### Effect of Intrinsic Reading Motivation and Gender on Reading Frequency

Descriptives for RF for each type of reading considering gender and each time of confinement are shown in Table 3.

**TABLE 3:** Means and SE of reading frequency (hours per day) per type of reading, time, and gender (untransformed data).

**Type of reading**	**Time**	**Gender**			
		**Female**		**Male**	
		***M***	***SE***	***M***	***SE***
Leisure	Before	0.93	0.02	0.94	0.03
	Beginning	1.27	0.02	1.25	0.03
	Month	1.42	0.02	1.30	0.03
News	Before	0.93	0.02	0.94	0.03
	Beginning	1.27	0.02	1.18	0.03
	Month	1.10	0.02	1.03	0.03
Socialize	Before	1.38	0.02	1.10	0.03
	Beginning	1.95	0.02	1.43	0.03
	Month	1.82	0.02	1.38	0.03
Study/Work	Before	1.88	0.02	1.81	0.03
	Beginning	2.07	0.02	2.15	0.03
	Month	2.14	0.02	2.14	0.03

*M, mean; SE, standard errors.*

**FIGURE 3:**
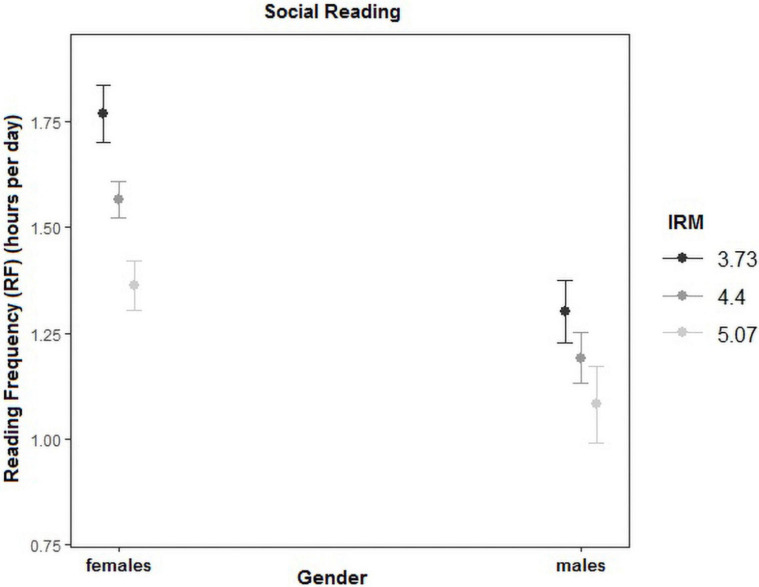
Interaction between reading frequency (RF), gender, and intrinsic reading motivation (IRM) for “Social Reading”.

The main effect of gender was significant only in the case of social reading, with females showing higher RF of social media than males (Mfemales = 1.89, *SE* = 0.02; Mmales = 1.4, *SE* = 0.03). Also, for this type of reading, the interaction between IRM and gender was significant, such that the slope of IRM was higher for females than for males (χ^2^ = 6.574; *p* = 0.01) (see Figure 3).

**FIGURE 4:**
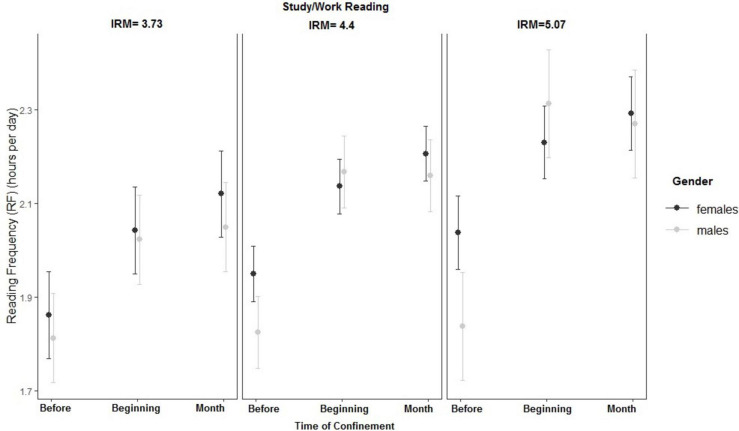
Interaction between reading frequency (RF), gender, and intrinsic reading motivation (IRM) for Study/Work Reading.

In the case of study/work reading, the three-way interaction between IRM, gender, and time was significant. As can be seen in Figure 4, females showed higher RF for study/work than males before the confinement, especially if they presented higher IRM. However, males only showed higher RF for work/study than females at the beginning of the confinement provided they presented high IRM.

### Effect of Distress, Intrinsic Reading Motivation, and Time of Confinement on Reading Frequency by Type of Reading

In order to explore H3, we split the participants into two groups according to the median of IRM (more IRM participants > 4.5, less IRM participants ≤ 4.5). We then conducted LMM analyses introducing distress and time of confinement as continuous factors, and RF as dependent variable (log-transformed in order to normalized it) per each type of reading and group of IRM (eight LMM analyses in total). Again, the participants were the random effect in each one of the eight models. The results of these analyses are shown in Table 4.

**TABLE 4:** Effects of distress and time on RF for each type of reading and higher and lower IRM.

**Study/Work**						
	**Sum sq**	**Mean sq**	**NumDF**	**DenDF**	***F* value**	**Pr(>F)**

**Higher IRM**						
Time	1.34	0.67	2	3903.1	6.09	0.002**
Distress	0.46	0.46	1	1973	4.17	0.041*
Time:distress	0.14	0.07	2	3903.1	0.65	0.520
**Lower IRM**						
Time	0.20	0.10	2	4400.7	0.93	0.393
Distress	0.03	0.03	1	2278	0.31	0.575
Time:distress	0.31	0.15	2	4400.7	1.42	0.241

**Leisure**						

	**Sum sq**	**Mean sq**	**NumDF**	**DenDF**	***F* value**	**Pr(>F)**

**Higher IRM**						
Time	1.86	0.93	2	3896.4	11.99	6.45E-06***
Distress	0.04	0.04	1	1893.5	0.52	0.471
Time:distress	0.34	0.17	2	3896.4	2.21	0.110
**Lower IRM**						
Time	0.61	0.30	2	4265	3.6	0.0275*
Distress	0.29	0.29	1	2129	3.44	0.064.
Time:distress	0.36	0.18	2	4265	2.14	0.118

**Social**						

	**Sum sq**	**Mean sq**	**NumDF**	**DenDF**	***F* value**	**Pr(>F)**

**Higher IRM**						
Time	2.49	1.24	2	3698.1	36.12	2.914E-16***
Distress	0.44	0.44	1	1844.5	12.91	0.000***
Time:distress	0.31	0.16	2	3698.1	4.55	0.011*
**Lower IRM**						
Time	1.59	0.8	2	4164.2	23.91	4.751E-11***
Distress	1.34	1.34	1	2122.4	40.29	2.669E-10***
Time:distress	0.07	0.03	2	4164.2	1.05	0.351

**News**						

	**Sum sq**	**Mean sq**	**NumDF**	**DenDF**	***F* value**	**Pr(>F)**

**Higher IRM**						
Time	0.36	0.18	2	3771.7	3.37	0.034*
Distress	0.06	0.06	1	1789.7	1.18	0.278
Time:distress	0.54	0.27	2	3771.7	5.08	0.006**
**Lower IRM**						
Time	0.29	0.15	2	4255	2.73	0.065.
Distress	0.07	0.07	1	2052.2	1.27	0.260
Time:distress	0.22	0.11	2	4255	2.06	0.128

*Sig. codes.* 0***; 0.001**; 0.01*. The data reported in this table have been calculated with transformed reading frequency (RF).

#### Study/Work Reading

For higher IRM participants, the effect of time was again significant, such that RF was significantly lower before of the confinement than at the beginning (z.ratio = −7.246; *p* < 0.001, β = −0.264) and at the month (z.ratio = −7.205; *p* < 0.001; β = −2.62) of confinement. However, the difference in RF was not significant between the beginning and the month of confinement (z.ratio = 0.04; *p* = 0.999). The effect of distress was also significant in such a way that the higher the distress, the lower the RF (β = −0.03).

For lower IRM participants, neither the effect of time nor the effect of distress was significant.

#### Leisure Reading

For higher IRM participants, the effect of time was significant again. In this type of reading, however, the difference in RF between the beginning and the month of confinement was also significant (z.ratio = −6.698; *p* < 0.001, β = −0.601), as in the case of before (z.ratio = −14.548; *p* < 0.001, β = −0.315) vs. beginning and before vs. month (z. ratio = −21.245; *p* < 0.001, β = −0.845). However, there was no main effect of distress for the higher IRM participants.

For lower IRM participants, the effect of time was significant again, such that RF was significantly lower before the confinement than at the beginning (z.ratio = −13.288; *p* < 0.001, β = −0.46) and at the month (z.ratio = −15.226; *p* < 0.0001; β = −0.527) of the confinement. However, the difference in RF was not significant between the beginning and the month of the confinement (z.ratio = −1.938; *p* = 0.128). As in the case of higher IRM, there was no main effect of distress for lower IRM.

#### Social Reading

**FIGURE 5:**
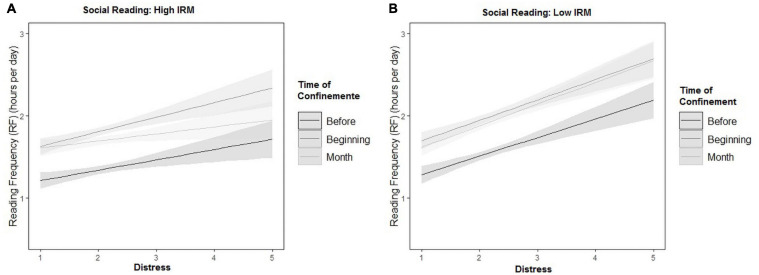
Interaction between reading frequency (RF), distress, and time of confinement for high intrinsic reading motivation (IRM) **(A)** and low IRM **(B)** for social reading.

For higher IRM participants, both the main effect of time (β = 0.42) and distress (β = 0.08) were significant. As it can be seen in Figure 5A, higher IRM participants showed more social RF at the beginning and after a month of the confinement than before and those participants more distressed showed higher RF of social media in general. However, the interaction between distress and time on RF was also significant, so the effect of distress was significant before (χ^2^ = 10.413, *p* < 0.004) and at the beginning of the confinement (χ^2^ = 19.144, *p* < 0.001) but not after a month (χ^2^ = 4.596, *p* = 0.096).

For lower IRF participants (Figure 5B), the main effect of time (β = 0.32) and distress (β = 0.12) were again significant. With regard to time, social RF was higher at the beginning and at the month of the confinement than before. Again, the higher the distress, the higher the RF for social media. The interaction time × distress was not significant.

#### News Reading

**FIGURE 6:**
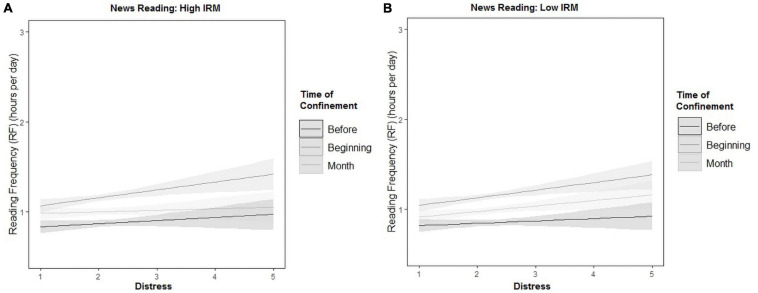
Interaction between reading frequency (RF), distress, and time of confinement for high intrinsic reading motivation (IRM) **(A)** and low IRM **(B)** for news reading.

In the case of higher IRM participants, the effect main effect of Time (β = 0.18) was significant but not the effect of distress. However, distress and time interacted significantly, so the effect of distress was significant at the beginning of the confinement (χ^2^ = 6.51, *p* < 0.01) but not before (χ^2^ = 10.358, *p* > 0.1) and after a month (χ^2^ = 0.183, *p* > 0.1) (see Figure 6A).

For lower IRM participants, neither the effect of distress nor the effect of time on RF was significant (see Figure 6B). The interaction Distress × Time was also not significant.

## Discussion

In this research, we used a correlational design to examine the relationship between IRM (as defined within SDT) and reading habits in an adult population taking into account types of reading (i.e., for leisure, for work or study, social networks, and news), gender, and distress generated by the exceptional situation of confinement in Spain caused by the COVID-19 pandemic. In line with theoretical expectations and previous studies, IRM was significantly associated with higher RF. This relationship was stronger for females and for leisure reading, and it was maintained despite the increased distress associated with confinement. Despite the exceptional nature of the pandemic and lockdown situation, this type of motivation seems to support the hypothesis of universal effects of intrinsic motivation, which are independent of contingencies (Ryan and Deci, 2019, 2020).

With regard to the first hypotheses, the main effect of IRM was significant, showing, as expected, that participants with higher IRM invested more hours reading (RF) than participants with lower IRM (Schiefele et al., 2012). This relationship held true at all time points: before, at the beginning, and after a month of confinement for leisure reading. The stronger relationships between IRM and RF were obtained for leisure, work, and social reading, although RF for each type of reading was different: more time invested for work reading, followed by social, and finally news and leisure at a similar level. Also, the relationship between IRM and RF was stronger for leisure than for work reading. This may mean that these types of reading could better satisfy competence and autonomy needs, that is why they are considered intrinsically motivating for the people who enjoy them (de Naeghel et al., 2012). Specifically, leisure reading seems to fit perfectly with SDT assumptions. People can initiate autonomously their reading behaviors even in more formal or compulsory reading contexts like study/work reading, meeting their needs from an intrinsic motivation. It is not totally surprising that those basic needs most positively related to IRM (autonomy and competence) are better satisfied by leisure and work/study reading. However, this effect was the weakest for news reading, at least in the confinement situation analyzed. The fact that news reading was not influenced that much by IRM could imply that different motives might be in play, for example, reading news may satisfy the person just for the feeling of being informed, not for reading itself.

Contrary to the expectations of the authors, the relationship between social reading and IRM was negative. It is possible that social reading (i.e., social media such as Instagram) facilitates a feeling of belonging in some situations (Pintrich and Schunk, 2006), but it may not be the most appropriate method of satisfying that need. In fact, all the participants reduced their social reading after a month of confinement. For individuals enjoying reading in itself, it seems logical to devote their reading time to leisure reading (which is the one with the strongest positive relation with IRM in this study), because leisure reading might be the most appropriate type of reading to satisfy the motivation of just reading, with no other purposes (like being informed or to connect with other people). Since time to read is somewhat limited in any given day, the more time a person spends in leisure reading, the less time s/he has left for other types of reading, which may explain why the higher IRM (implying longer leisure reading time), the shorter time devoted to social reading. However, this is just a speculative hypothesis that needs to be confirmed or falsified in future studies. Also, SDT recognizes that most intentional behaviors can be multiply motivated (Litalien et al., 2017). In this sense, people who did social reading may have been simultaneously motivated by several types of regulations at the same time (Ryan and Deci, 2020), for example, intrinsic and identified. Social reading may satisfy less self-determined needs, or it may satisfy those basic needs in a different way than leisure reading.

Regarding the second hypothesis, although reading frequencies were higher for females in general, the only significant main effect of type of reading and gender was for social reading. Females were more intrinsically motivated to read than men, as also demonstrated by other studies (Ratelle et al., 2007; Vansteenkiste et al., 2009). In this regard, previous research has suggested the possibility that women with higher autonomous motivation could be more efficient using their time and environment to focus on their studies (e.g., Vallerand et al., 1997). This can explain why women showed higher RF for study/work than men before the confinement. However, males showed higher RF for work/study than females at the beginning of the confinement provided they presented high IRM. This is very interesting, since it suggests that gender differences in reading frequency may be influenced by the context (like a confinement situation).

Finally, intrinsic reading motivation seems to play a different role in reading behavior when the interaction with distress and type of reading is included. Thus, when individuals have high intrinsic motivation, this type of motivation seems to protect reading behavior only in the case of leisure reading. The results reveal in this sense that it is necessary to consider the interaction between distress and the content of the different readings in order to understand the changes in reading behavior despite high intrinsic motivation. Thus, in the case of study/work reading, distress seems to affect the decrease in reading frequency even if individuals have high intrinsic motivation. In this case, it is possible to think that the interaction between distress and the content of these readings somehow overrides the weight of intrinsic motivation as far as reading behavior is concerned.

In social and news reading, people with high intrinsic motivation read more when distress came into play. In the case of social reading, this interaction can be explained by the potential that social media can become addictive in stressful situations like the COVID pandemic (Zhao and Zhou, 2021). With regard to news reading, at the same level of motivation and stress, timing also seems to have an impact on reading behavior, especially understandable in cases like the one experienced during the past confinement. Thus, it is possible that, at the beginning of the confinement, people were more motivated to read the news because of the need to perceive some control or autonomy in a context in which they were beginning to lack it. This effect ceases to be observed as the confinement progresses.

Therefore, distress seems to pose a differential impact depending on the type of reading: when the reading activity does not pose a demand or requirement (e.g., social or study/work reading) and is not related to stressful stimuli (e.g., news reading), intrinsic reading motivation is a protector against distress and facilitates the satisfaction of personal basic needs. However, when the type of reading is more demanding, distress has a negative impact, and paradoxical events may happen, such as the results obtained showing how the higher IRM participants for study/work reading devote less time to that type of reading if they are distressed, probably because the association of work/study with distress does not allow to satisfy the basic needs associated with that type of reading.

These results support the argument of the need for studying these variables beyond an educational context (Howard et al., 2021) and a young population. Reading motivation should be very present in educational research, but it is now undeniable that we should expand the scope to adult populations in other contexts, since there might be important effects for distress and well-being that may help different interventions or screening strategies. This also suggests that developing intrinsic motivation to read since early childhood may be important not only to promote reading *per se* and for the well-known positive impact on learning and performance but also for the effects beyond the educational context related to the health and well-being of individuals.

### Limitations and Further Research

The non-probabilistic sampling procedure and the correlational design are the main limitations of this study. The great majority of the readers involved in this sample are young adults with a medium to high education level. The findings of this study, thus, cannot be generalized to adult readers in general and do not allow us to establish causal relations between variables. It would be interesting to investigate how this stressful environment affected the reading habits of children, too, or if those habits were influenced or mediated by the behaviors of parents. Also, educational level is a critical factor, which did not receive sufficient attention in this study. In addition, socio-economic status might be a critical variable modulating the effects of motivation to read on reading habits.

Because of the limitations of the confinement, all measures collected in this, as well as in other studies on the recent pandemic, are based on self-reports. Objective reading measures could yield different results and, in general, could be useful to validate the findings of this study. Given the exceptional circumstances of this pandemic, replicating these findings in other studies may be difficult. However, further studies can be conducted in situations in which individuals experience a temporary condition of distress. Exploring how intrinsic reading motivation relates to non-academic behaviors, such as well-being, may lead to interesting findings. It would be desirable to perform more controlled studies exploring all motivational dimensions and how each of them (and their combinations) relates to different types of reading.

## Conclusion

Intrinsic motivation behaviors (as such, self-determined) have a positive impact during mandatory lockdown periods. It is important to highlight that this confinement was known to be only temporary, but there was also uncertainty at some points about the specific duration. In this sense, people who showed higher IRM managed to protect their reading habits regardless of a stressful context.

Intrinsic reading motivation seems to maintain its positive impact even in extreme situations, such as the global pandemic. However, not every type of reading should be used to satisfy the basic needs associated with IRM. As this study shows, in some cases, a high IRM is related to a higher RF but also to higher distress. When reading involves demands or requirements from the reader and it is performed in a stressful environment like the recent confinement, the relationship between IRM and RF may not be necessarily positive, since increased RF can be associated with higher distress and, therefore, with lower wellbeing. This has been the case for social reading.

These results contribute to the field and to those people interested in motivation and its relationship with the adaptive behaviors of individuals. Results also support previous findings of the importance of consciously promoting this type of motivation from an early age not only because of the benefits for learning and academic performance but also for the impact beyond the educational context on the management of difficult or stressful situations of individuals.

## Data Availability Statement

Publicly Available datasets were analyzed in this study. This data can be found here: https://osf.io/24et3/?view_only=68613c73dd71499bbdadbad93d4ca79a.

## Ethics Statement

Ethical review and approval was not required for the study on human participants in accordance with the local legislation and institutional requirements. The patients/participants provided their written informed consent to participate in this study.

## Author Contributions

RD, IF, and AM implemented the study. IF conducted the statistical analyses. RD, ÁJ, and AM drafted the manuscript. MR, MG-S, and FN formatted the study. RD, ÁJ, AM, BA, and JR reviewed the study and performed substantial suggestions. All the authors participated in the conception of the study, reviewed the manuscript critically for relevant intellectual content, and approved the submitted version.

## Conflict of Interest

The authors declare that the research was conducted in the absence of any commercial or financial relationships that could be construed as a potential conflict of interest.
